# Inoculation of α-synuclein preformed fibrils into the mouse gastrointestinal tract induces Lewy body-like aggregates in the brainstem via the vagus nerve

**DOI:** 10.1186/s13024-018-0257-5

**Published:** 2018-05-11

**Authors:** Norihito Uemura, Hisashi Yagi, Maiko T. Uemura, Yusuke Hatanaka, Hodaka Yamakado, Ryosuke Takahashi

**Affiliations:** 1 Department of Neurology, Kyoto University Graduate School of Medicine, 54 Shogoin-Kawaharacho, Kyoto, Sakyoku 606-8507 Japan; 2 Center for Research on Green Sustainable Chemistry, Tottori University, 4-101 Koyamacho-minami, Tottori, Tottori 680-8550 Japan

**Keywords:** Parkinson’s disease, α-synuclein, Lewy bodies, Propagation, Enteric nervous system, Vagus nerve

## Abstract

**Background:**

Intraneuronal α-synuclein (α-Syn) aggregates known as Lewy bodies (LBs) and the loss of dopaminergic neurons in the substantia nigra pars compacta (SNpc) are the pathological hallmarks of Parkinson’s disease (PD). Braak’s hypothesis based on autopsy studies suggests that Lewy pathology initially occurs in the enteric nervous system (ENS) and then travels retrogradely to the dorsal motor nucleus of the vagus nerve (dmX), proceeding from there in a caudo-rostral direction. Recent evidence that α-Syn aggregates propagate between interconnected neurons supports this hypothesis. However, there is no direct evidence demonstrating this transmission from the ENS to the dmX and then to the SNpc.

**Methods:**

We inoculated α-Syn preformed fibrils (PFFs) or phosphate-buffered saline (PBS) into the mouse gastric wall and analyzed the progression of the pathology.

**Results:**

The mice inoculated with α-Syn PFFs, but not with PBS, developed phosphorylated α-Syn (p-α-Syn)–positive LB-like aggregates in the dmX at 45 days postinoculation. This aggregate formation was completely abolished when vagotomy was performed prior to inoculation of α-Syn PFFs, suggesting that the aggregates in the dmX were retrogradely induced via the vagus nerve. Unexpectedly, the number of neurons containing p-α-Syn–positive aggregates in the dmX decreased over time, and no further caudo-rostral propagation beyond the dmX was observed up to 12 months postinoculation. P-α-Syn–positive aggregates were also present in the myenteric plexus at 12 months postinoculation. However, unlike in patients with PD, there was no cell-type specificity in neurons containing those aggregates in this model.

**Conclusions:** These results indicate that α-Syn PFF inoculation into the mouse gastrointestinal tract can induce α-Syn pathology resembling that of very early PD, but other factors are apparently required if further progression of PD pathology is to be replicated in this animal model.

**Electronic supplementary material:**

The online version of this article (10.1186/s13024-018-0257-5) contains supplementary material, which is available to authorized users.

## Background

Parkinson’s disease (PD) is the most common neurodegenerative movement disorder [1]. The pathologic findings include loss of dopaminergic neurons in the substantia nigra pars compacta (SNpc) and the presence of α-synuclein (α-Syn) aggregates in the form of Lewy bodies (LBs) and Lewy neurites [2]. Clinically, PD is characterized by four cardinal motor manifestations: bradykinesia, muscle rigidity, resting tremor, and postural instability [3]. Other symptoms include various nonmotor features such as hyposmia, constipation, anxiety, depression, orthostatic hypotension, urinary dysfunction, rapid eye movement sleep behavior disorder, and cognitive dysfunction. Some of them are known as prodromal symptoms of PD or risk factors for developing the disease [4, 5].

Braak et al. systematically analyzed the pathology in cases with incidental LB pathology or sporadic PD and suggested that Lewy pathology initially develops in the olfactory bulb and the dorsal motor nucleus of the vagus nerve (dmX) and then spreads in the brain stereotypically [6, 7]. Moreover, since Lewy pathology is found in the enteric nervous system (ENS) in the early stage of PD, they hypothesized that Lewy pathology in the ENS travels retrogradely to the dmX and then proceeds from there in a caudo-rostral direction [8]. Braak et al. integrated these observations into a staging system for PD consisting of six stages, each defined by Lewy pathology found in particular neuroanatomical structures. This staging system has gained much attention because it seems to explain the clinical course of PD well, from prodromal symptoms appearing early, to motor symptoms in the middle stage, and finally to cognitive dysfunction in the late stage [3].

Braak et al. proposed that an unknown neurotropic pathogen such as a virus initiates the pathogenesis underlying PD to explain the development of Lewy pathology in two independent sites: the olfactory bulb and the ENS [9]. In this scenario, the neurotropic pathogen is taken up by neurons and then progresses within nervous system by way of axonal transport and transsynaptic transmission. The observation that healthy neurons transplanted in the brains of patients with PD gradually developed LBs also suggested that certain pathogenetic events in PD are not cell-autonomous and raised the possibility that α-Syn may be the propagating agent [10, 11]. Afterwards animal studies have demonstrated that misfolded fibrillar forms of α-Syn self-propagate and spread between interconnected neurons in the central nervous system (CNS), suggesting that cell-to-cell transmission of pathological proteins contributes to PD progression [12, 13].

Despite the great impact of the Braak’s hypothesis on investigation of the clinicopathologic progression of PD, this hypothesis is still widely debated, with several issues needing resolution. For instance, it remains unclear whether Lewy pathology in the ENS indeed spreads to the dmX, and, if so, how it travels on from the dmX to the SNpc [14, 15]. A previous study demonstrated that different forms of α-Syn (i.e., PD brain lysate, monomeric, oligomeric, and fibrillar α-Syn) inoculated into the rat intestinal wall traveled retrogradely in the vagus nerve and were found in dmX neurons at 6 days postinoculation [16]. However, it was not shown whether aggregated α-Syn pathology formed in neurons to which inoculated α-Syn has been transported [16]. Another study found that virus-mediated α-Syn overexpression in the dmX and the ambiguous nucleus induced caudo-rostral progression of α-Syn–positive neuritic pathology, but not somatic LB-like α-Syn pathology [17].

Therefore, to address the issues described above, we inoculated α-Syn preformed fibrils (PFFs) into the gastrointestinal tract of wild-type mice and observed the chronological progression of the pathology. We demonstrated that α-Syn PFFs inoculated into the mouse gastric wall induced phosphorylated α-Syn (p-α-Syn)–positive LB-like aggregates in both the ENS and dmX. Further chronological analysis provides insights into the pathogenesis and progression of PD.

## Methods

### Preparation of recombinant α-Syn monomers and preformed fibrils

Mouse α-Syn PFFs were generated as described previously with minor modifications [18]. *Escherichia coli* BL21 (DE3) (BioDynamics Laboratory) were transformed with plasmid pRK172 encoding the mouse α-Syn cDNA sequence and incubated in LB medium to an optical density of 0.3 at 600 nm. α-Syn expression was induced by 0.1 mM isopropyl β-D-1-thiogalactopyranoside for 4 h. The bacteria were pelleted by centrifugation at 4000 g at 4 °C for 5 min and lysed by freeze/thaw and sonication. The lysate was clarified by boiling for 5 min, followed by centrifugation at 20,400 g at 4 °C for 15 min. The supernatant was subjected to ion exchange using Q sepharose fast flow (GE Healthcare), and α-Syn was precipitated with 50% (% saturation) ammonium sulfate. Purified α-Syn was dialyzed against dialysis buffer (150 mM KCl, 50 mM Tris-HCl, pH 7.5) and cleared by ultracentrifugation at 186,000 g at 4 °C for 20 min. The protein concentration was determined with a Pierce BCA Protein Assay kit (Thermo Fisher). Purified α-Syn was diluted in dialysis buffer containing 0.1% (w/v) NaN_3_ to 7 mg/ml, followed by incubation at 37 °C in a shaking incubator (AS ONE, SI-300C) at 1000 rpm for 10 days. The α-Syn PFF pellet was obtained by ultracentrifugation at 186,000 g at 20 °C for 20 min and stored at − 80 °C until use. The pellet was dissolved in 8 M guanidine hydrochloride to determine the protein concentration with a Pierce BCA Protein Assay kit (Thermo Fisher). The α-Syn PFFs in phosphate-buffered saline (PBS) (2 μg/μl) were sonicated with an ultrasonic wave disruption system (Cosmo Bio, Bioruptor) for 2 min before inoculation into the mouse gastric wall.

### Sodium dodecyl sulfate-polyacrylamide gel electrophoresis and western blot analysis

For sodium dodecyl sulfate (SDS)-polyacrylamide gel electrophoresis (PAGE), sample buffer (1% [w/v] SDS, 12.5% [w/v] glycerol, 0.005% [w/v] bromophenol blue, 2.5% [v/v] 2-mercaptoethanol, 25 mM Tris-HCl, pH 6.8) was added to α-Syn monomer solution or the α-Syn PFF pellet, which was resuspended by vortex. Samples containing 10 μg protein were loaded in each lane and separated on 12% (w/v) gels for SDS-PAGE. For Coomassie Brilliant Blue (CBB) staining, gels were incubated in CBB staining solution (0.1% [w/v] PhastGel Blue R-350 [GE Healthcare], 30% [v/v] methanol, 10% [v/v] acetic acid) at room temperature. For western blot analysis, proteins were transferred to polyvinylidene difluoride membranes with a Trans-Blot SD Semi-Dry Transfer Cell (Bio-Rad). The membranes were treated with 0.4% (w/v) paraformaldehyde (PFA) in PBS for 30 min at room temperature before blocking with 5% skim milk to prevent detachment of α-Syn from the blotted membranes [19]. The membranes were incubated with an anti-α-Syn primary antibody (BD Transduction, #610787 [Syn-1], 1:2000) at 4 °C overnight, followed by reaction with horseradish peroxidase-conjugated secondary antibodies (Santa Cruz, #sc-2005, 1:5000) for 1 h at room temperature. Immunoreactive bands were detected with Pierce ECL Western Blotting Substrate (Thermo Fisher), and the chemiluminescent signal was detected with an Amersham Imager 600 imager (GE Healthcare).

### Thioflavin T assay

α-Syn monomers or PFFs, 7 μg each, were incubated in 250 μl of 5 μM Thioflavin T (Sigma-Aldrich, #T3516) solution for 15 min at room temperature. The fluorescence at 535 nm (excitation 450 nm) was measured with a multi-label plate reader (PerkinElmer, 2030 ARVO X). Thioflavin T solution alone was measured as a blank.

### Transmission electron microscopy

α-Syn PFFs, 5 μg, were placed on a 400-mesh carbon-coated copper grid (Nissin EM). The excess solution was removed with filter paper after the sample had stood for 1 min. The PFFs adsorbed on the grid were negatively stained with a 2% (w/v) uranyl acetate solution. Electron micrographs were acquired using a transmission electron microscope (JEOL, JEM-1400 Plus) at 80 kV.

### Animals and ethics statement

C57BL/6J male mice at 2 months of age were used for the present study (*n* = 61). All experimental procedures used in this study followed national guidelines. The Animal Research Committee of Kyoto University granted ethical approval and permission (MedKyo 17,184).

### Inoculation of α-Syn PFFs into the mouse gastric wall

Mice were anesthetized with Avertin (1.875% [w/v] 2,2,2-tribromoethanol, 1.25% [v/v] 3-methyl-1-butanol). A 2-cm incision was made in the abdominal midline, followed by inoculation of α-Syn PFFs (*n* = 48) or PBS (*n* = 3) into the gastric wall. Among the mice inoculated with α-Syn PFFs, 8 mice underwent right cervical vagotomy just prior to inoculation of α-Syn PFFs. Each of 8 sites was inoculated with 3 μl of α-Syn PFFs in PBS (2 μg/μl) or with PBS using a 37 gauge needle (Saito Medical Instruments) fitted to 10 μl syringe (Hamilton, #701LT).

### Cervical vagotomy

Following anesthesia, a 1-cm incision was made at the midline of the mouse neck. The right vagus nerve was identified between the common carotid artery and the jugular vein behind the submandibular gland. After isolation from the carotid sheath, the vagus nerve was cut with a pair of tweezers.

### FluoroGold verification of vagotomy

Verification of vagotomy using FluoroGold was performed as described with modifications [20]. Five days prior to sacrifice, unvagotomized or vagotomized mice (3 mice per group) were injected intraperitoneally with 1.2 mg of hydroxystilbamidine (AAT Bioquest) in 1 ml of saline. Frozen sections were obtained as described below and observed with a BZ-× 710 fluorescence microscope (KEYENCE).

### Histologic and immunohistochemical analysis

Frozen and paraffin sections were used for histologic and immunohistochemical analysis. Following perfusion with 4% (w/v) PFA in PBS, the brains were removed and immersed in 4% (w/v) PFA in PBS. For frozen sections, the brains were replaced in 15% (w/v) sucrose in PBS and subsequently in 30% (w/v) sucrose in PBS at 4 °C each overnight. The brains were embedded in Surgipath FSC 22 (Leica), and 10-μm sections were obtained with a CM1950 cryostat (Leica). For paraffin sections, the mouse brains were dehydrated and embedded in paraffin, and 8-μm paraffin sections were prepared with a HM 325 rotary microtome (MICROM). For immunohistochemical analysis, the following primary antibodies were used: anti–α-Syn (BD Transduction, #610787 [Syn-1], 1:1000), anti–p-α-Syn (Abcam, #ab51253 [EP1536Y], 1:10000), anti–p-α-Syn (Wako, #015-25191 [#64], 1:2000), anti–p-α-Syn (Abcam, #ab184674 [81A], 1:5000), anti-nitrated α-Syn (Santa Cruz, #sc-32,279 [Syn514], 1:200), anti-choline acetyltransferase (ChAT) (Millipore, #AB144P, 1:1000), anti-p62 (MBL, #PM045, 1:1000), anti-ubiquitin (DAKO, #Z0458, 1:500), anti-vasoactive intestinal polypeptide (VIP) (Abcam, #ab8556, 1:50), anti-nitric oxide synthase 1 (NOS1) (Santa Cruz, #A-11, 1:200), and anti-substance P (Millipore, #MAB356, 1:200). The sections were incubated at 4 °C with primary antibodies for 2 d and then processed for visualization. As secondary antibodies, Histofine (Nichirei Bioscience) was used for diaminobenzidine staining, and Alexa Fluor 488 or 594-conjugated antibodies (Molecular Probes) for immunofluorescence. For p-α-Syn and Thioflavin S (ThS, Santa Cruz, #sc-391005) double-labeling staining, after immunolabeled with p-α-Syn antibody, slides were incubated with 0.05% ThS in 50% ethanol followed by differentiation with 80% ethanol. For assessment of p-α-Syn pathology, every 10^th^ paraffin section was stained with anti–p-α-Syn antibody (EP1536Y). To assess ChAT-positive neurons in the dmX, every 10^th^ paraffin section was stained with anti-ChAT antibody. The numbers of p-α-Syn–positive and ChAT-positive neurons were manually counted. Sections were examined with a BX43 microscope (Olympus), a BZ-X710 fluorescence microscope (KEYENCE), and an FV-1000 confocal laser scanning microscope (Olympus).

### Statistical analysis

One-way ANOVA with Tukey’s post-hoc test was used. Statistical calculations were performed with GraphPad Prism Software, Version 5.0.

## Results

### Inoculation of α-Syn preformed fibrils into the mouse gastric wall

**Fig. 1:**
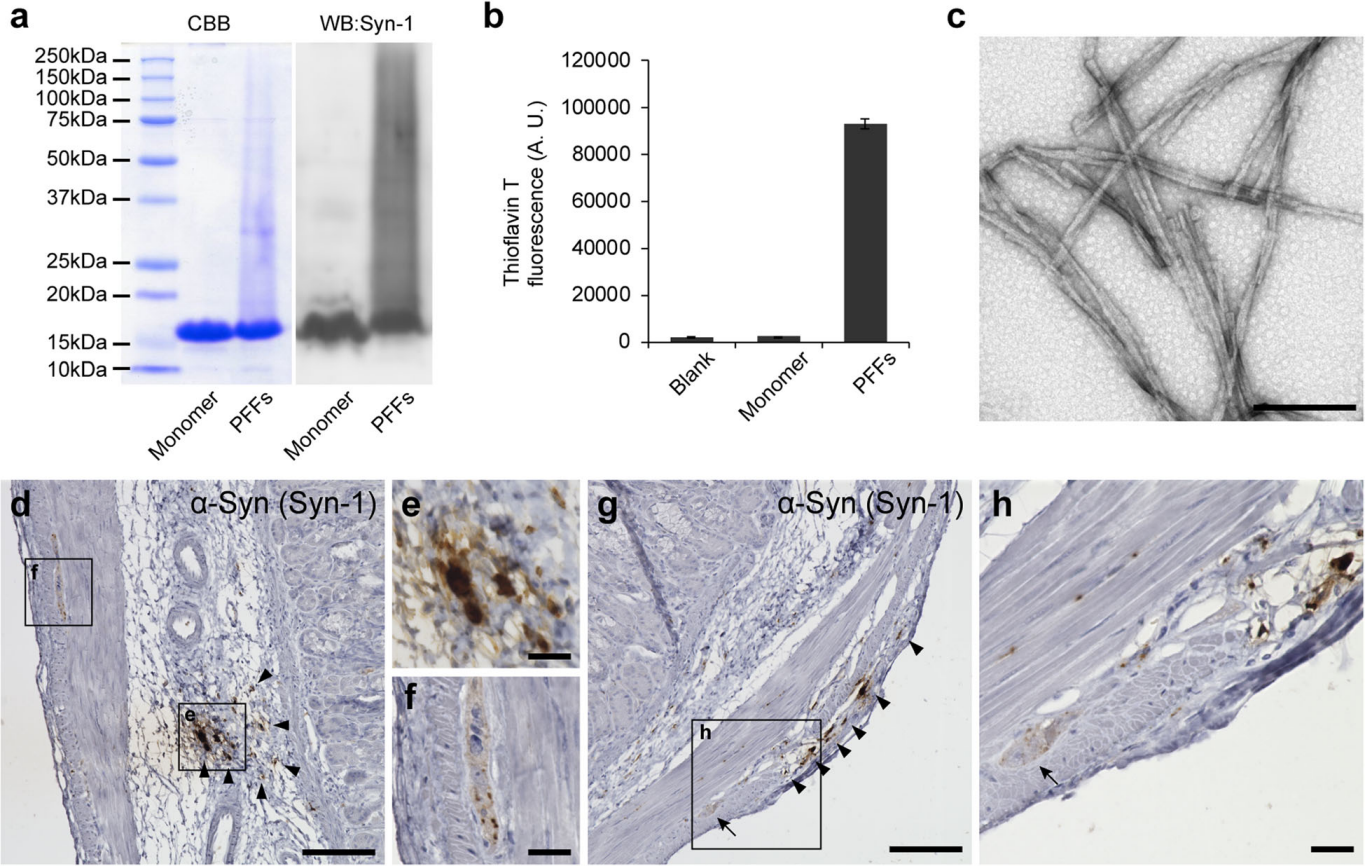
Inoculation of α-synuclein (α-Syn) preformed fibrils (PFFs) into the mouse gastric wall. **a** Coomassie brilliant blue (CBB) staining and western blot (WB) analysis of α-Syn. Smear bands are observed in PFFs. **b** Thioflavin T assay. High-intensity fluorescence at 535 nm is detected in PFFs (*n* = 3). A. U., arbitrary unit. **c** Transmission electron microscopy of α-Syn PFFs. Scale bar 200 nm. **d**-**h** α-Syn immunohistochemistry in the stomach at 45 days postinoculation. **d** α-Syn deposition in the submucosa (arrowheads). Scale bar 100 μm. **e** High-magnification image of α-Syn deposition in the submucosa. Scale bar 20 μm. **f** High-magnification image of neurons in the myenteric plexus. Scale bar 20 μm. **g** α-Syn deposition in the muscular layers. Neurons in the myenteric plexus (arrow). Scale bar 100 μm. **h** High-magnification image of α-Syn deposition in the muscular layers. Scale bar 20 μm. Data are the mean ± SEM

We used mouse rather than human α-Syn PFFs in this study because mouse α-Syn PFFs are more potent for inducing α-Syn pathology and its propagation as compared with human α-Syn PFFs inoculated in wild-type mouse brains [13, 21]. First, we generated mouse α-Syn PFFs and validated their characteristics. SDS-PAGE followed by CBB staining or western blotting showed smear bands of α-Syn PFFs, suggesting α-Syn polymerization (Fig. 1a). High-intensity Thioflavin T fluorescence of α-Syn PFFs indicated that α-Syn PFFs comprised β-sheet–rich structures (Fig. 1b). Transmission electron microscopy revealed fibrillar structures in the α-Syn PFFs (Fig. 1c). We inoculated α-Syn PFFs or PBS into the gastric wall of the mice. To confirm that the PFFs properly inoculated into the gastric wall, we immunostained paraffin sections of the gastric wall with anti-α-Syn antibody. Deposition of inoculated α-Syn PFFs was found in the submucosal layer (Fig. 1d, e) and in the muscular layer (Fig. 1g, h) even 45 days after α-Syn PFFs inoculation. α-Syn immunohistochemistry also showed α-Syn–positive dots around the myenteric neurons, some of which are efferent vagus nerve terminals (Fig. 1f) [20].

### Phosphorylated α-Syn pathology was observed in the dmX 45 days after α-Syn PFF inoculation

**Fig. 2:**
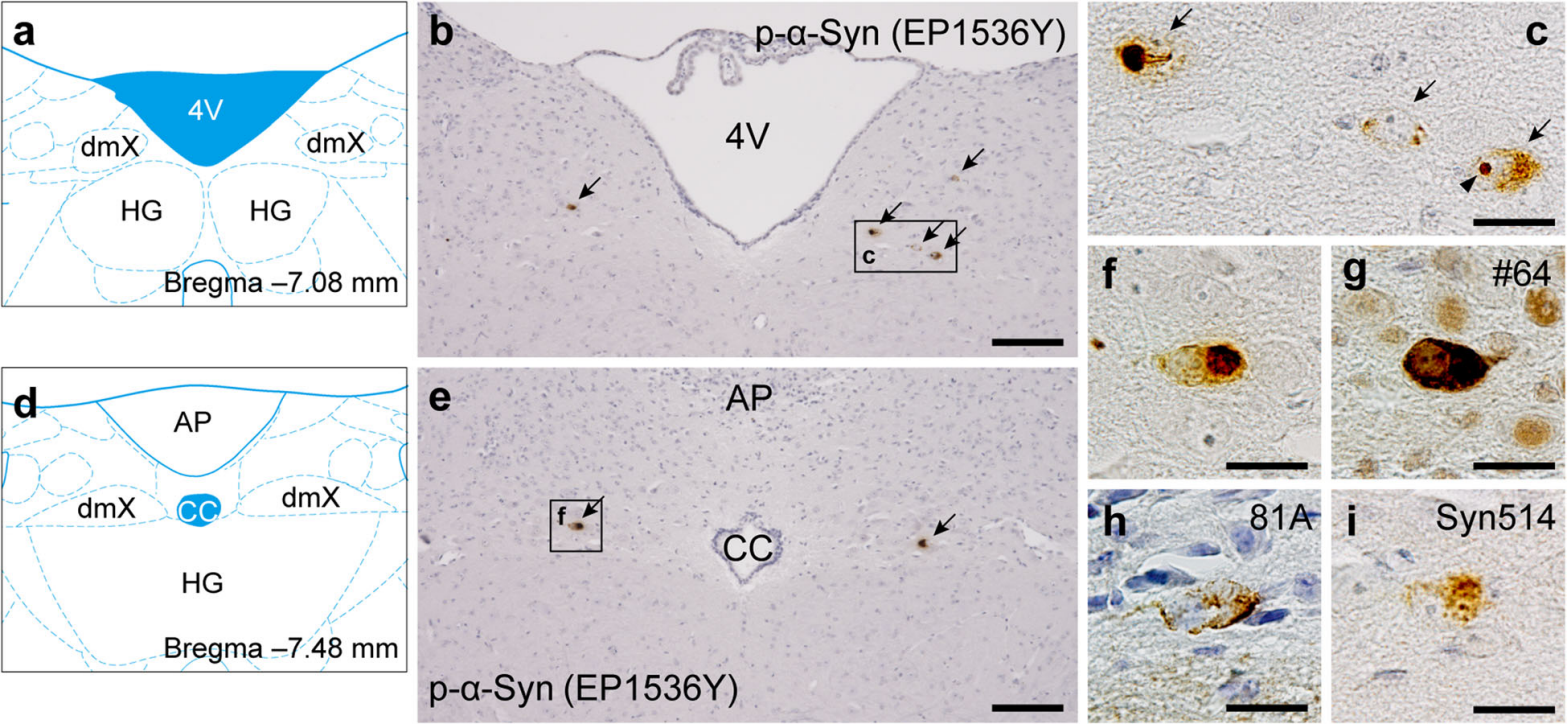
Phosphorylated α-synuclein (p-α-Syn) pathology in the dmX 45 days after inoculation of α-Syn preformed fibrils. **a** Schematic of anatomy at bregma − 7.08 mm modified from [47]. dmX, dorsal motor nucleus of the vagus nerve; HG, hypoglossal nucleus; 4V, fourth ventricle. **b** P-α-Syn (EP1536Y) immunohistochemistry around bregma − 7.08 mm. Scale bar 100 μm. **c** High-magnification image of p-α-Syn–positive cells (arrows). P-α-Syn–positive intranuclear dots are occasionally observed (arrowhead). Scale bar 20 μm. **d** Schematic of anatomy at bregma − 7.48 mm. AP, area postrema; cc, central canal. **e** P-α-Syn (EP1536Y) immunohistochemistry around bregma − 7.48 mm. Scale bar 100 μm. **f** High-magnification image of p-α-Syn–positive cells. Scale bar 20 μm. **g**–**i** Immunohistochemistry assessing p-α-Syn (#64), p-α-Syn (81A), or nitrated α-Syn (Syn514) shows p-α-Syn–positive or nitrated α-Syn–positive cells in the dmX. Scale bar 20 μm

Forty-five days after inoculation of α-Syn PFFs into the gastric walls, we examined the brains and stomachs of the mice. Immunostaining for p-α-Syn, a marker of human Lewy pathology, showed p-α-Syn–positive neurons in both rostral and caudal parts of the dmX in mice that had been inoculated with α-Syn PFFs (Fig. 2a–f). All those neurons had p-α-Syn in the cytoplasm, whereas a few also exhibited intranuclear p-α-Syn–positive dots (Fig. 2c). We confirmed that the pathology in the dmX were detectable with other anti–p-α-Syn antibodies and an anti-nitrated α-Syn antibody (Fig. 2g–i) [22]. However, p-α-Syn pathology were rarely observed in the stomachs 45 days after α-Syn PFF inoculation (Additional file 1: Figure S1), nor were they seen in either brains or stomachs of mice inoculated with only PBS (Additional file 2: Figure S2).

### Phosphorylated α-Syn pathology exhibited LB-like features

**Fig. 3:**
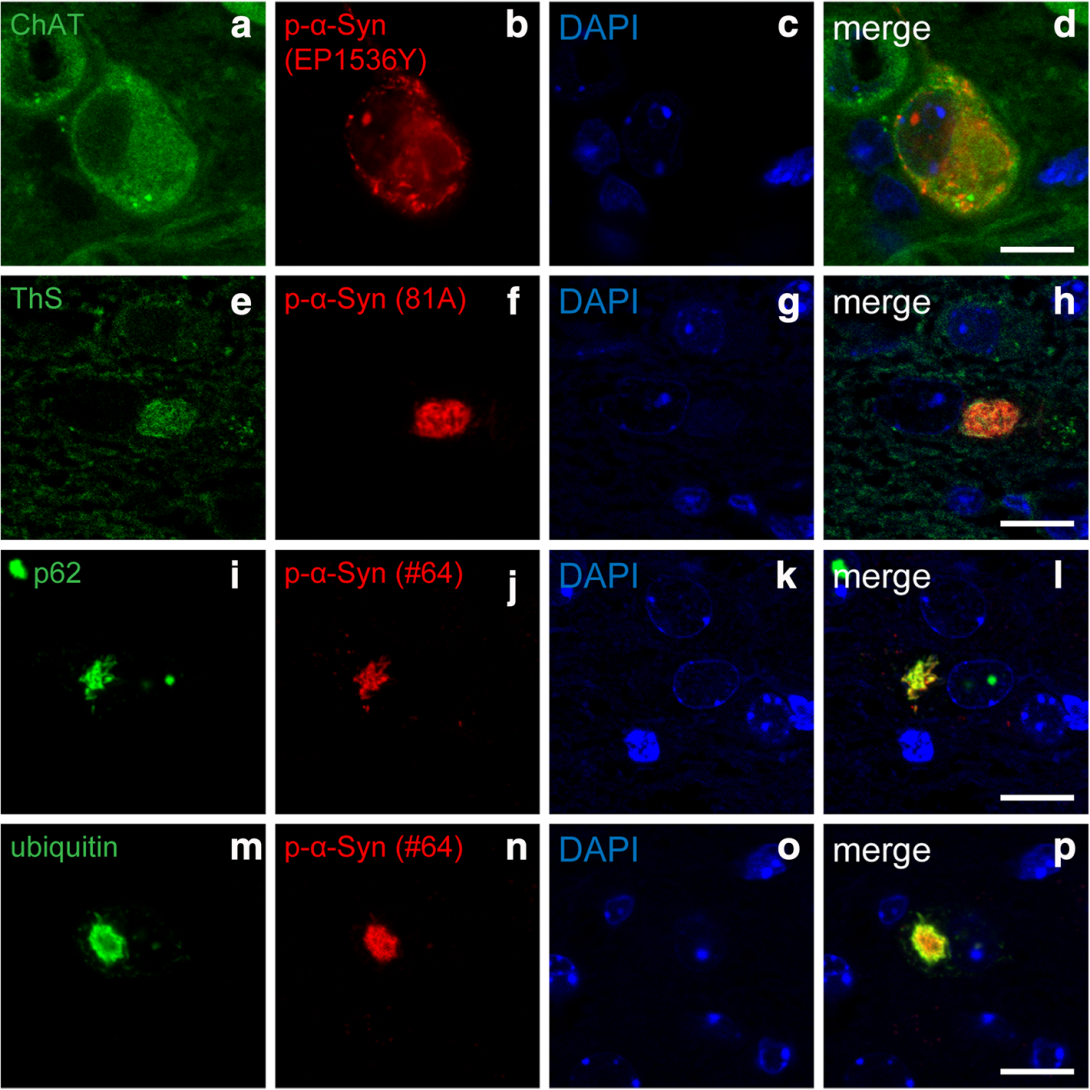
Characterization of p-α-Syn pathology in the dmX 45 days after inoculation of α-Syn preformed fibrils. **a**–**d** Double immunostaining for choline acetyltransferase (ChAT) (green) and p-α-Syn (EP1536Y, red). Scale bar 10 μm. **e**–**h** Thioflavin S (ThS) staining (green) and immunostaining for p-α-Syn (81A, red). Scale bar 10 μm. **i**–**l** Double immunostaining for p62 (green) and p-α-Syn (#64, red). Scale bar 10 μm. **m**–**p** Double immunostaining for ubiquitin (green) and p-α-Syn (#64, red). Scale bar 10 μm

Next, we examined the pathological features of the p-α-Syn pathology in the dmX. We confirmed that it was present in ChAT-positive neurons of the dmX (Fig. 3a–d). The p-α-Syn pathology was positive for ThS, indicating that they were composed of β-sheet–rich amyloid fibrils (Fig. 3e–h). Most of the p-α-Syn pathology was also immunopositive for p62 (Fig. 3i–l) and ubiquitin (Fig. 3m–p), both of which are LB markers. All these observations indicated that the p-α-Syn pathology in the dmX shared properties in common with the LBs seen in patients with PD.

### Vagus nerve mediated the formation of p-α-Syn pathology in the dmX

**Fig. 4:**
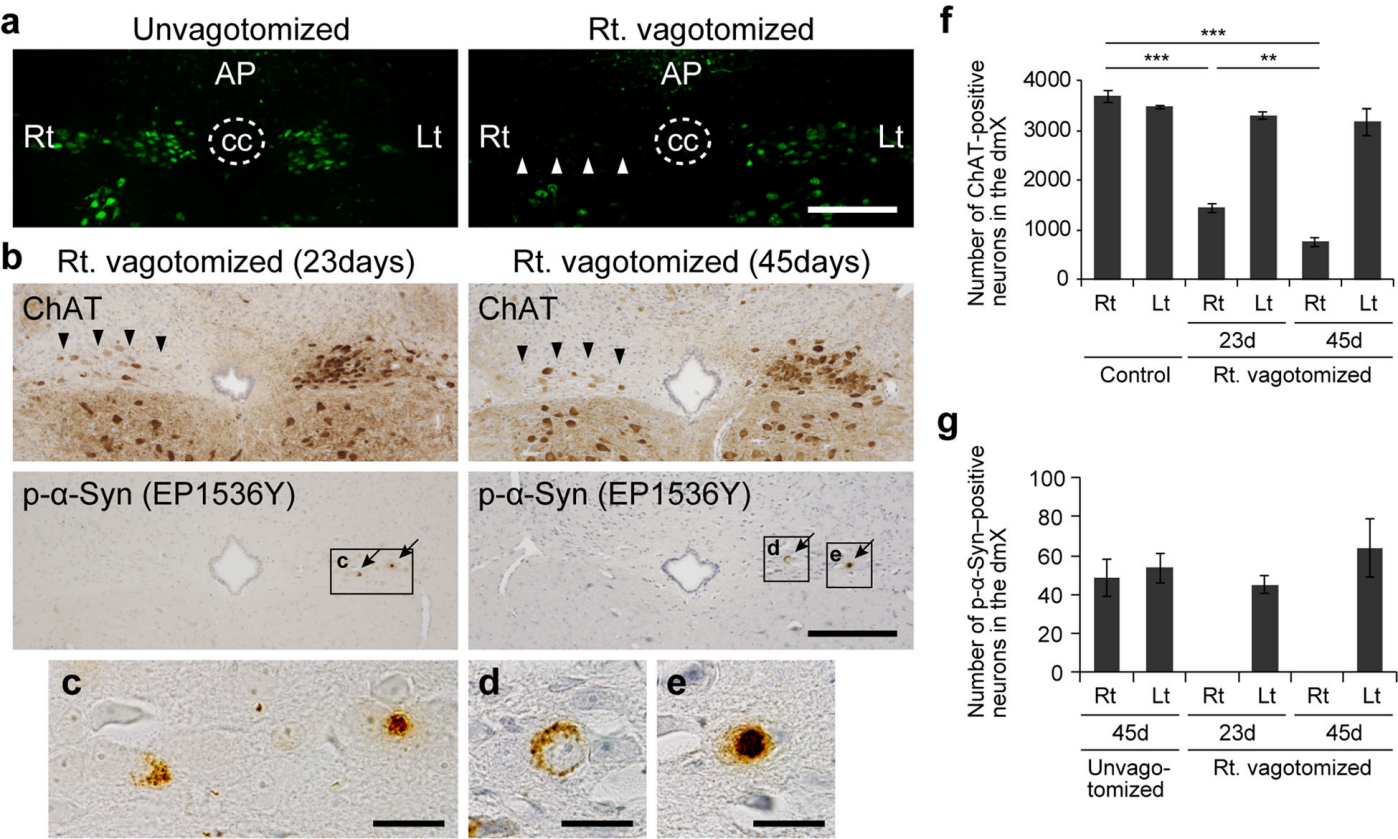
Vagotomy prevents appearance of p-α-Syn pathology in the dmX after inoculation of α-Syn preformed fibrils. **a** FluoroGold labeling of the dmX. Left panel: Labeling appears in the dmX bilaterally 5 days after intraperitoneal injection of FluoroGold in unvagotomized mice. Right panel: FluoroGold labeling is absent in the right dmX when right (Rt.) vagotomy was performed prior to intraperitoneal injection of FluoroGold. AP, area postrema; cc, central canal. Scale bar 200 μm. **b** choline acetyltransferase (ChAT) and p-α-Syn immunohistochemistry in right-vagotomized mice 23 days and 45 days after α-Syn PFF inoculation. The number of ChAT-positive neurons in the right dmX decreased at 23 days and further decreased at 45 days (arrowheads). P-α-Syn–positive aggregates are present only in the left dmX at both time points (arrows). Scale bar 200 μm. **c**–**e** High-magnification images of p-α-Syn–positive cells. Scale bars 20 μm. **f** Number of ChAT-positive neurons in the dmX of untreated mice (Control) and right-vagotomized mice 23 days and 45 days after α-Syn PFF inoculation (*n* = 3–4). One-way ANOVA with Tukey’s multiple-comparisons test was performed; **p < 0.01, ***p < 0.001. **g** Number of p-α-Syn–positive neurons in the dmX of unvagotomised mice 45 days after α-Syn PFF inoculation and right-vagotomized mice 23 days and 45 days after α-Syn PFF inoculation (*n* = 4). Data are the mean ± SEM

Anatomically, it was presumed that α-Syn PFFs inoculated into the gastric wall retrogradely induced p-α-Syn–positive LB-like aggregates in the dmX via the vagus nerve. To verify this, we inoculated α-Syn PFFs into the gastric wall immediately after hemivagotomy. We confirmed that vagotomy was successful using a retrograde neuronal tracer, FluoroGold. Intraperitoneal injection of FluoroGold labeled neurons in the dmX as has been reported in rats (Fig. 4a) [20]. When FluoroGold was injected after hemivagotomy, neurons on the vagotomy side were not labeled, indicating that the vagotomy had succeeded (Fig. 4a). However, it was reported that vagotomy causes neuronal loss over time in the murine dmX on the vagotomy side [23], so we analyzed some vagotomized mice 23 days after α-Syn PFF inoculation in addition to 45 days. The numbers of ChAT-positive neurons on the vagotomy side decreased to 40% and to 20% compared with those of untreated mice 23 days and 45 days after surgery, respectively (Fig. 4b, f). Although a certain number of ChAT-positive neurons survived on the vagotomy side, p-α-Syn–positive neurons were observed only on the unvagotomized side at both 23 and 45 days (Fig. 4b–e, g). This suggests that the vagus nerve indeed was the pathway by which α-Syn PFFs inoculated into the stomach resulted in formation of p-α-Syn aggregates in the dmX.

### Phosphorylated α-Syn–positive neurons decreased in number, and no further propagation was observed up to 12 months after α-Syn PFFs inoculation

**Fig. 5:**
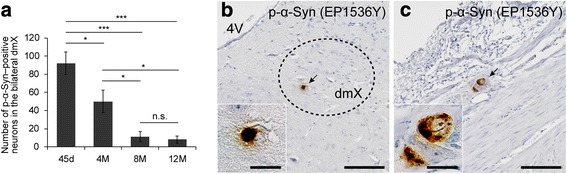
Chronological analysis of p-α-Syn pathology after inoculation of α-Syn preformed fibrils. **a** Number of p-α-Syn–positive neurons in the dmX of mice bilaterally examined at 45 days, 4 months, 8 months, and 12 months postinoculation of α-Syn preformed fibrils (*n* = 5–8). One-way ANOVA with Tukey’s multiple-comparisons test was performed; *p < 0.05, ***p < 0.001, n.s. means not significant. **b** p-α-Syn (EP1536Y) immunohistochemistry in the dmX of a mouse at 12 months postinoculation. Dashed line outlines the approximate area of the dmX. dmX, dorsal motor nucleus of the vagus nerve; 4V, fourth ventricle. Scale bar 100 μm. A p-α-Syn–positive neuron (arrow) is enlarged in the inset. Scale bar 20 μm. **c** p-α-Syn (EP1536Y) immunohistochemistry in the myenteric plexus of a mouse at 12 months postinoculation. Scale bar 100 μm. A p-α-Syn–positive neuron (arrow) is enlarged in the inset. Scale bar 20 μm. Data are the mean ± SEM

Then, we analyzed p-α-Syn pathology up to 12 months after α-Syn PFF inoculation. Unexpectedly, the number of p-α-Syn–positive neurons in the dmX bilaterally decreased over time (Fig. 5a). No further propagation of p-α-Syn pathology beyond the dmX was observed during this period. At 12 months postinoculation, there were only a small number of p-α-Syn–positive aggregates in the dmX, which appeared as denser inclusions than those observed at 45 days postinoculation (Fig. 5b). There were also a few p-α-Syn–positive neurons in the myenteric plexus at 12 months postinoculation (Fig. 5c). We also examined the thoracic spinal cord but observed no p-α-Syn pathology up to 12 months postinoculation (Additional file 3: Figure S3).

### No apparent cell-type specificity in neurons containing p-α-Syn–positive aggregates in the myenteric plexus

**Fig. 6:**
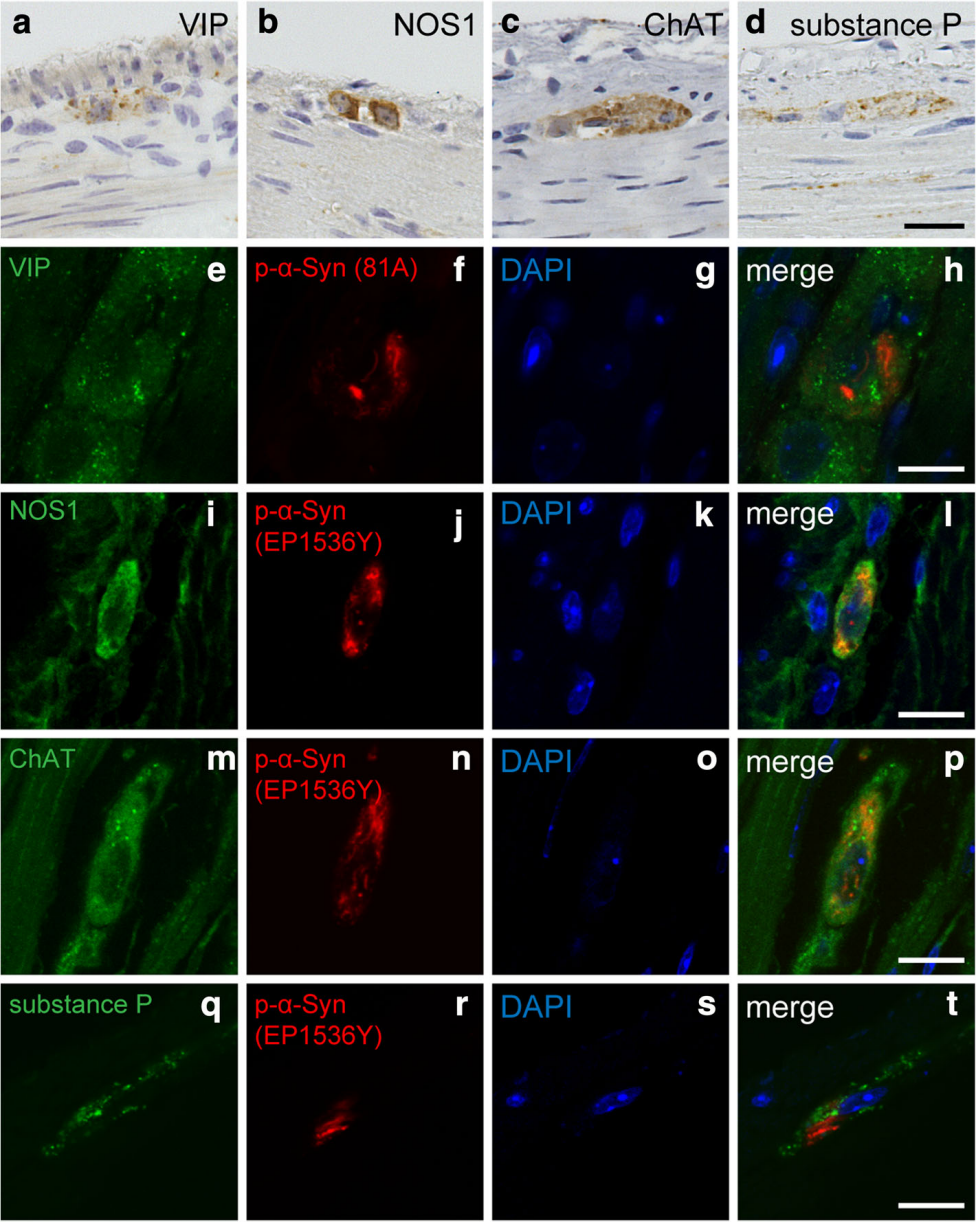
Analysis of types of neurons containing p-α-Syn–positive aggregates in the myenteric plexus. **a**-**d** Representative images of vasoactive intestinal peptide (VIP), nitric oxide synthase 1 (NOS1), choline acetyltransferase (ChAT), and substance P immunohistochemistry in the myenteric plexus. **e**–**h** Double immunostaining for VIP (green) and p-α-Syn (81A, red). Scale bar 10 μm. **i**–**l** Double immunostaining for NOS1 (green) and p-α-Syn (EP1536Y, red). Scale bar 10 μm. **m**–**p** Double immunostaining for ChAT (green) and p-α-Syn (EP1536Y, red). Scale bar 10 μm. **q**–**t** Double immunostaining for Substance P (green) and p-α-Syn (EP1536Y, red). Scale bar 10 μm

An autopsy study of patients with PD reported that most LBs in the ENS are present in VIP-positive neurons [24]. According to Braak’s hypothesis, Lewy pathology initially occurs in the ENS, suggesting that a specific type of neurons in the ENS may play a key role in the pathogenesis of PD. Finally, we therefore examined which types of myenteric neurons contained LB-like aggregates 12 months after α-Syn PFF inoculation. Although single markers cannot completely distinguish the various types of neurons, many VIP-positive neurons in the myenteric plexus are inhibitory motor neurons and are also immunopositive for NOS1 (Fig. 6a, b) [25, 26]. Excitatory motor neurons are immunopositive for ChAT and substance P (Fig. 6c, d). We conducted double immunofluorescent analysis using antibodies against these markers along with anti–p-α-Syn antibodies. A substantial portion of p-α-Syn aggregates were present in VIP- or NOS1-positive neurons (83% [10/12] and 67% [6/9], respectively; Fig. 6e–l), but some aggregates were also present in ChAT- or substance P-positive neurons (45% [6/13] and 33% [4/12], respectively; Fig. 6m–t). We therefore did not find cell-type specificity in neurons containing p-α-Syn–positive aggregates in the myenteric plexus in this mouse model.

## Discussion

A number of studies in both humans and animals have been conducted after Braak et al. published the “gut-to-brain” hypothesis, but controversy remains. In an whole-body autopsy study of several hundred cases, there was not a single case in which Lewy pathology was present in the ENS but not in the CNS, which argues against Braak’s hypothesis [27]. On the other hand, two independent cohort studies reported that the risk of developing PD may be substantially decreased following truncal vagotomy, which does support the hypothesis by suggesting the involvement of the vagus nerve in the pathogenesis of PD [28, 29]. In the present study, we investigated the progression of LB-like pathology as proposed by Braak’s hypothesis by inoculating α-Syn PFFs into the mouse gastric wall. Although the experimental conditions differ from the putative progression of Lewy pathology from the ENS to the dmX in human PD, we demonstrated that misfolded fibrillar forms of α-Syn present in the gastric wall were capable of inducing LB-like pathology in the dmX via the vagus nerve. Unexpectedly, however, the number of neurons in the dmX containing p-α-Syn–positive aggregates decreased over time. It may be caused by the death of those neurons [12, 30] or chronological degradation of those aggregates.

According to the Braak’s staging system, the neuroanatomical structures affected at stages 1, 2, and 3 include the dmX, locus coeruleus (LC), and SNpc, respectively. However, though brainstem nuclei are interconnected with each other directly or indirectly, it remains unclear what routes Lewy pathology follow from the dmX, through the LC, to the SNpc in the human brainstem [15]. Identifying such routes is indispensable for a better understanding of the clinicopathologic progression of PD, including the development of prodromal symptoms. Because the dmX receives input from a broad range of brain regions such as the solitary nucleus, LC, raphe nuclei, hypothalamus, and amygdala [31], we expected to find p-α-Syn–positive aggregates in those structures as well. However, no p-α-Syn–positive aggregates were observed in any mouse brain region other than the dmX up to 12 months postinoculation. A recent study reported that α-Syn PFF inoculation into the rat descending colon induced a small number of p-α-Syn–positive aggregates in the LC as well as dmX at 1 month postinoculation [32]. However, the study also reported that no p-α-Syn–positive aggregates were found anywhere in the rat brain, including the dmX and LC, at later time points [32].

There are several possible interpretations of our observation that p-α-Syn pathology did not spread beyond the dmX. First, some genetic or environmental factors may be required for further propagation, as sporadic PD is thought to be caused by the interaction of such factors. Indeed, it was shown that intracerebral inoculation of α-Syn PFFs or tau fibrils induced spreading of α-Syn or tau aggregates in interconnected brain regions of wild-type mice but, over time, the number of affected brain regions peaked [33, 34]. These findings suggest that seeding pathological aggregates in wild-type mice is not sufficient to replicate natural disease progression, indicating that some additional factors are required for continuous spread of aggregates. For example, single nucleotide polymorphisms in *SNCA* (the α-Syn gene) which increase α-Syn expression have been identified as a genetic risk factor for PD [35]. *Snca-*null mice never show LB-like pathology or propagation of α-Syn pathology when α-Syn PFFs are inoculated into their brains [12], so it is conceivable that the opposite situation, that is, transgenic or virus-mediated overexpression of α-Syn, might accelerate the formation and propagation of α-Syn pathology. Mutations in *GBA* (the glucocerebrosidase gene), the strongest genetic risk factor for PD, as well as aging, one of the environmental risk factors, both affect the proteolytic activity of the autophagy-lysosomal pathway, possibly promoting the formation and propagation of α-Syn pathology [36, 37]. Both the environmental neurotoxin rotenone and the inflammogen lipopolysaccharide have been reported to induce intraneuronal α-Syn accumulation in mouse brains, suggesting that they might also facilitate the propagation of α-Syn pathology [38–40]. Therefore, including some of these factors along with α-Syn PFF inoculation may enable us to replicate the progression of PD pathology in accordance with Braak’s hypothesis.

Second, the α-Syn PFFs used in this study may not have been potent enough to induce neuron-to-neuron propagation. Recent evidence has documented that recombinant α-Syn monomers can form α-Syn aggregates with a variety of conformations and biological activities [41]. Distinct α-Syn aggregates named “strains” are associated with different pathologies and have variable seeding activity, cell toxicity, and cell-type preference both *in vitro* and *in vivo* [42, 43]. Importantly, distinct α-Syn strains maintain their intrinsic conformations and properties even in seeded fibrillization reactions *in vitro* and *in vivo* [43, 44]. These suggest that distinct α-Syn strains probably have varying potency for inducing neuron-to-neuron propagation in the mouse brains. We occasionally observed uncommon pathologies in PD, p-α-Syn–positive dots in the neuronal nuclei in the dmX and p-α-Syn–positive aggregates in ChAT- or substance P-positive neurons in the ENS. These pathological features of this mouse model might be derived from different properties of α-Syn PFFs from those of α-Syn aggregates in PD. It may be worth trying to use other α-Syn strains or α-Syn aggregates purified from PD brains.

Finally, given that there are specific neural routes vulnerable to spread of α-Syn aggregates, it may be that caudo-rostral routes originating from the dmX are less likely to be among those specific routes. Based on a CNS-wide survey of several hundred autopsy cases, Beach et al. proposed a unified staging system for LB diseases [45]. In this system, stage I is defined as involvement of only the olfactory bulb, whereas the brain stem is involved in later stages, II or III. It was reported that there are almost no cases of incidental LB pathology without olfactory bulb involvement as well as many that involve the olfactory bulb alone [27]. The authors hypothesized that α-Syn pathology in peripheral organs emerges after that in the CNS because peripheral α-Syn pathology was not observed in stage I [27]. A previous study demonstrated that virally overexpressed α-Syn in the rat midbrain gained access into dmX neurons and then reached presynaptic terminals of the vagus nerve in the gastric wall, supporting this hypothesis [46]. Based on these observations and hypotheses, we may have to consider a rostro-caudal pathway as well as a caudo-rostral pathway of α-Syn spread in future research.

## Conclusions

In conclusion, this study demonstrated that inoculation of α-Syn PFFs into the mouse gastric wall can induce LB-like pathology in the dmX via the vagus nerve, supporting Braak’s “gut-to-brain” hypothesis. However, further caudo-rostral spread of α-Syn pathology was not observed up to 12 months postinoculation. Other factors may be required for further progression of PD pathology beyond the dmX. Our chronological, detailed analysis revealed similarities and dissimilarities of this mouse model to human PD and provides important clues for designing future research.

## Additional Files

Additional file 1: **Figure S1.** Phosphorylated α-synuclein (p-α-Syn) pathology in the stomach 45 days after α-Syn preformed fibrils were inoculated into a mouse gastric wall. **a** P-α-Syn (EP1536Y) immunohistochemistry of the serial section from Fig. 1d. No apparent p-α-Syn pathology is seen. Scale bar 100 μm. **b** P-α-Syn (EP1536Y) immunohistochemistry of the serial section from Fig. 1g. No apparent p-α-Syn pathology is seen in the myenteric neurons. Scale bar 100 μm. **c** P-α-Syn (EP1536Y) immunohistochemistry of another section showing p-α-Syn aggregates in the myenteric plexus (arrow), enlarged in the inset. Scale bar 20 μm. (TIF 4963 kb)

Additional file 2: **Figure S2.** No phosphorylated α-synuclein (p-α-Syn) pathology is seen in either mouse brain or stomach 45 days after phosphate-buffered saline inoculation into a mouse gastric wall. **a** P-α-Syn (EP1536Y) immunohistochemistry of a section around bregma − 7.08 mm. Scale bar 100 μm. 4V, fourth ventricle. **b** P-α-Syn (EP1536Y) immunohistochemistry of a section around bregma − 7.48 mm. Scale bar 100 μm. AP, area postrema; cc, central canal. **c** P-α-Syn (EP1536Y) immunohistochemistry in the stomach. Scale bar 100 μm. (TIF 7863 kb)

Additional file 3: **Figure S3.** No phosphorylated α-synuclein (p-α-Syn) pathology in the thoracic spinal cord 12 months after inoculation of α-Syn preformed fibrils into the mouse gastric wall. **a** P-α-Syn (EP1536Y) immunohistochemistry of the thoracic spinal cord. Scale bar 100 μm. (TIF 3586 kb)

## Abbreviations

4V: fourth ventricle
α-Syn: α-synuclein
A. U.: arbitrary unit
AP: Area postrema
CBB: Coomassie Brilliant Blue
cc: central canal
ChAT: Choline acetyltransferase
CNS: Central nervous system
dmX: dorsal motor nucleus of the vagus nerve
ENS: Enteric nervous system
HG: Hypoglossal nucleus
LB: Lewy body
NOS1: Nitric oxide synthase 1
PAGE: Polyacrylamide gel electrophoresis
PBS: Phosphate buffer saline
PBS: Phosphate-buffered saline
PD: Parkinson’s disease
PFA: Paraformaldehyde
PFF: Preformed fibril
p-α-Syn: phosphorylated α-synuclein
SDS: Sodium dodecyl sulfate
ThS: Thioflavin S
VIP: Vasoactive intestinal polypeptide
WB: western blot
