# Correction to: Inoculation of α-synuclein preformed fibrils into the mouse gastrointestinal tract induces Lewy body-like aggregates in the brainstem via the vagus nerve

**DOI:** 10.1186/s13024-019-0331-7

**Published:** 2019-07-26

**Authors:** Norihito Uemura, Hisashi Yagi, Maiko T. Uemura, Yusuke Hatanaka, Hodaka Yamakado, Ryosuke Takahashi

**Affiliations:** 1 Department of Neurology, Kyoto University Graduate School of Medicine, 54 Shogoin-Kawaharacho, Kyoto, Sakyoku 606-8507 Japan; 2 Center for Research on Green Sustainable Chemistry, Tottori University, 4-101, Koyamacho-minami, Tottori, Tottori 680-8550 Japan

**Correction to: Mol Neurodegener**

**https://doi.org/10.1186/s13024-018-0257-5**

The original article [1] mistakenly omitted essential information regarding Fig. 1c; thus, the authors would like to note that Fig. 1c describes transmission electron microscopy of α-Syn PFFs **before** sonication.
