# Supplementary figures and images for: Inoculation of α-synuclein preformed fibrils into the mouse gastrointestinal tract induces Lewy body-like aggregates in the brainstem via the vagus nerve
Source: Mol Neurodegener. 2018 May 11;13:21. doi: 10.1186/s13024-018-0257-5 (PMC5948849; doi:10.1186/s13024-018-0257-5)

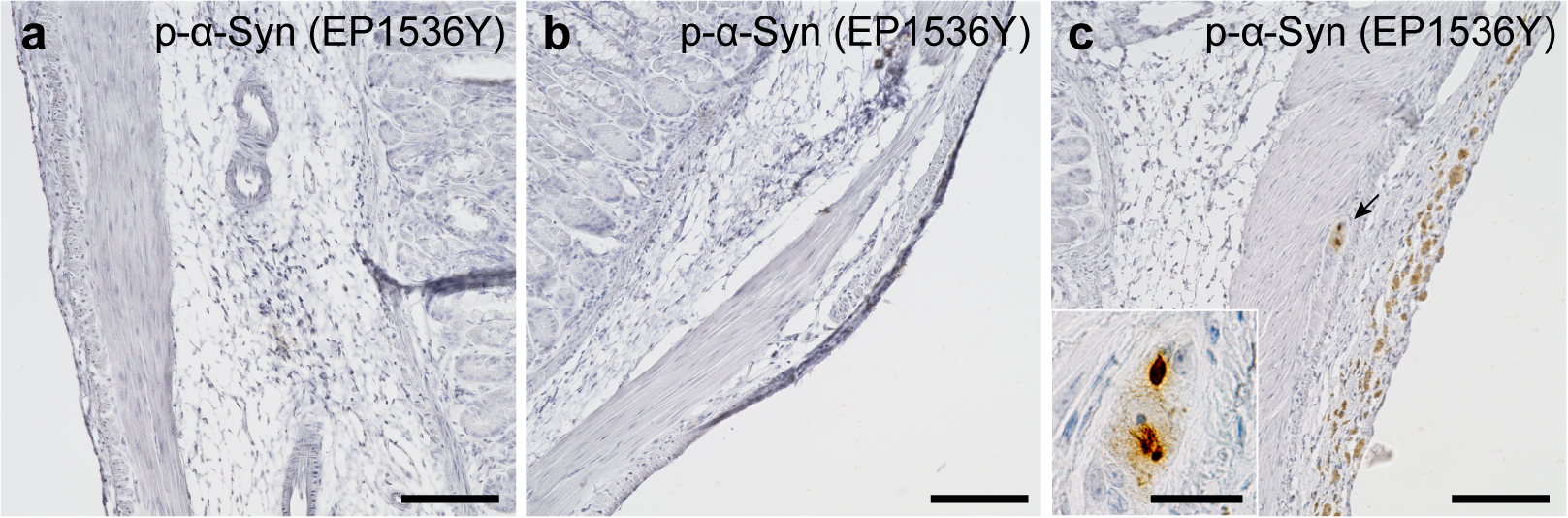

Supplement: Supplementary file 1 — Figure S1. Phosphorylated α-synuclein (p-α-Syn) pathology in the stomach 45 days after α-Syn preformed fibrils were inoculated into a mouse gastric wall. a P-α-Syn (EP1536Y) immunohistochemistry of the serial section from Fig. 1d. No apparent p-α-Syn pathology is seen. Scale bar 100 μm. b P-α-Syn (EP1536Y) immunohistochemistry of the serial section from Fig. 1g. No apparent p-α-Syn pathology is seen in the myenteric neurons. Scale bar 100 μm. c P-α-Syn (EP1536Y) immunohistochemistry of another section showing p-α-Syn aggregates in the myenteric plexus (arrow), enlarged in the inset. Scale bar 20 μm. (TIF 4963 kb) [file 13024_2018_257_MOESM1_ESM.tif]

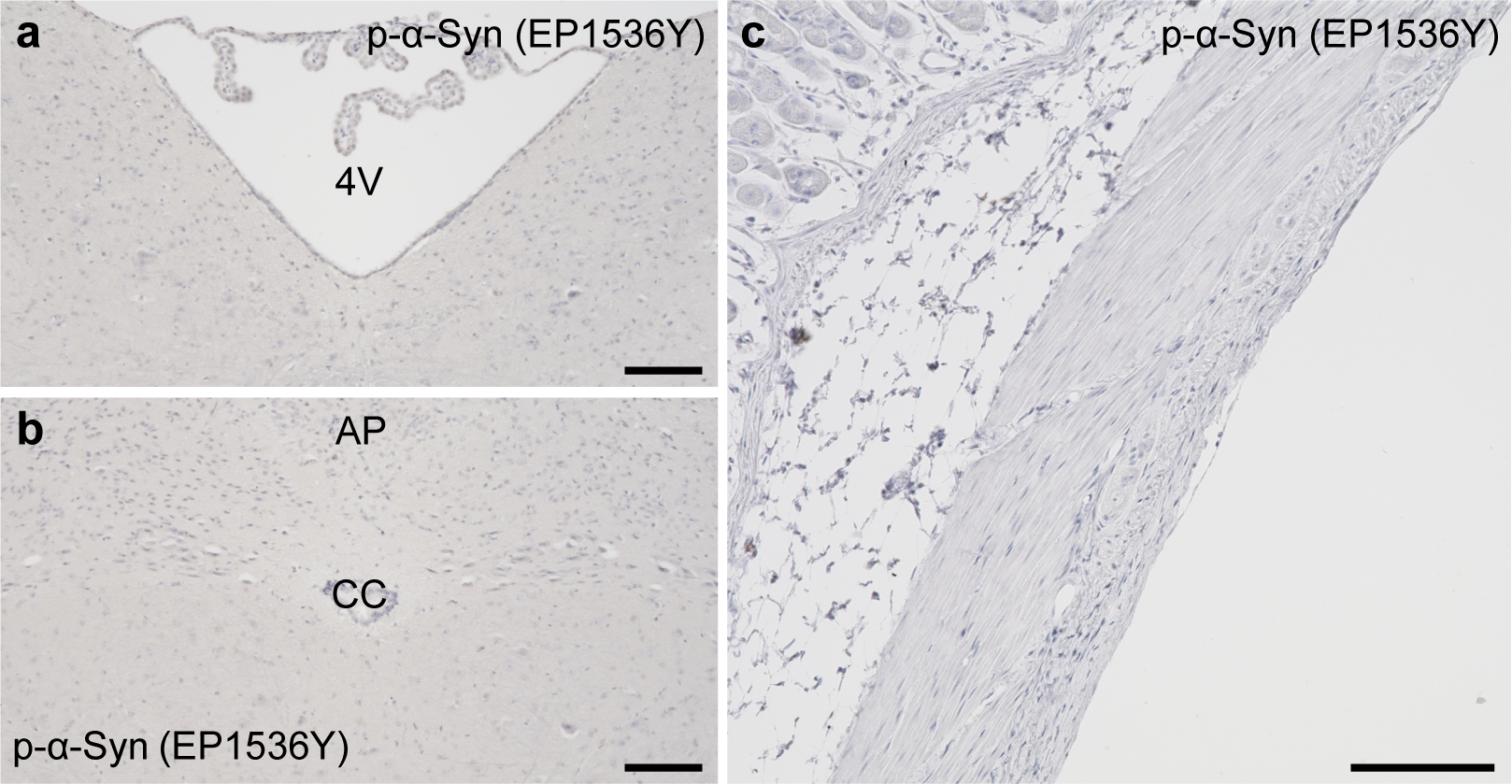

Supplement: Supplementary file 2 — Figure S2. No phosphorylated α-synuclein (p-α-Syn) pathology is seen in either mouse brain or stomach 45 days after phosphate-buffered saline inoculation into a mouse gastric wall. a P-α-Syn (EP1536Y) immunohistochemistry of a section around bregma − 7.08 mm. Scale bar 100 μm. 4V, fourth ventricle. b P-α-Syn (EP1536Y) immunohistochemistry of a section around bregma − 7.48 mm. Scale bar 100 μm. AP, area postrema; cc, central canal. c P-α-Syn (EP1536Y) immunohistochemistry in the stomach. Scale bar 100 μm. (TIF 7863 kb) [file 13024_2018_257_MOESM2_ESM.tif]

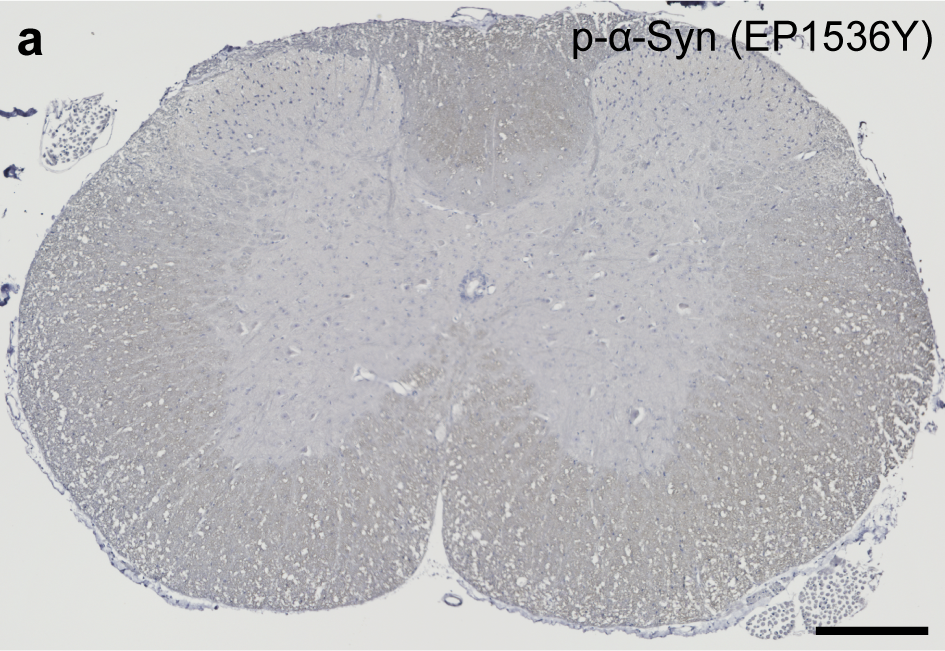

Supplement: Supplementary file 3 — Figure S3. No phosphorylated α-synuclein (p-α-Syn) pathology in the thoracic spinal cord 12 months after inoculation of α-Syn preformed fibrils into the mouse gastric wall. a P-α-Syn (EP1536Y) immunohistochemistry of the thoracic spinal cord. Scale bar 100 μm. (TIF 3586 kb) [file 13024_2018_257_MOESM3_ESM.tif]
